# Effects of Gender, Sterilization, and Environment on the Spatial Distribution of Free-Roaming Dogs: An Intervention Study in an Urban Setting

**DOI:** 10.3389/fvets.2020.00289

**Published:** 2020-05-27

**Authors:** Saulo Nascimento de Melo, Eduardo Sergio da Silva, David Soeiro Barbosa, Rafael Gonçalves Teixeira-Neto, Gustavo Augusto Lacorte, Marco Aurélio Pereira Horta, Diogo Tavares Cardoso, Guilherme Loureiro Werneck, Claudio José Struchiner, Vinícius Silva Belo

**Affiliations:** ^1^Campus Centro Oeste Dona Lindu, Universidade Federal de São João del Rei, Divinópolis, Brazil; ^2^Instituto de Ciências Biológicas, Universidade Federal de Minas Gerais, Belo Horizonte, Brazil; ^3^Instituto Federal de Educação, Ciência e Tecnologia Minas Gerais, Campus Bambuí, Bambuí, Brazil; ^4^Fundação Oswaldo Cruz, Instituto Oswaldo Cruz, Rio de Janeiro, Brazil; ^5^Departamento de Epidemiologia, Instituto de Medicina Social, Universidade Do Estado Do Rio de Janeiro, Rio de Janeiro, Brazil; ^6^Fundação Getúlio Vargas, Escola de Matemática Aplicada, Rio de Janeiro, Brazil

**Keywords:** free-roaming dogs, surgical sterilization, dog behavior, urban areas, spatial analysis, zoonosis spread

## Abstract

Information concerning the factors affecting the circulation and distribution of free-roaming dogs is crucial in developing control actions and limiting the spread of zoonoses. The present study analyzes the influence of gender, sterilization, and environment on the spatial distribution of free-roaming dogs in urban settings. Animals were captured/recaptured in seven consecutive morning sampling efforts conducted at 2-monthly intervals in control and intervention areas in a medium-size town in southeastern Brazil. Capture locations were georeferenced and captured animals were microchipped before being released at their original capture sites. Dogs captured in the intervention area were subjected additionally to surgical sterilization prior to release. Home range (HR) areas were calculated by applying the minimum convex polygon method to dogs that had been captured at least three times. Land coverage zones were determined from satellite images and overlaid on maps of the study areas along with the locations of 22 commercial food outlets. HR areas showed a global mean of 448 m^2^ and a median of 28 m^2^, values that were smaller than those reported previously for dogs in rural regions. The median HR of females (64.m^2^) was higher than that of males (15 m^2^), while median HRs of animals in the control and intervention areas were similar (27 and 28.5 m^2^, respectively). Variability of HR was high, although animals with small HRs predominated. Free-roaming dogs grouped primarily in urbanized and transitional regions, and their spatial distribution was positively correlated with locations of commercial food outlets. While sterilization did not influence HR size, the search for food was a key factor in determining mobility and spatial aggregation of free-roaming dogs. Our findings are pertinent in understanding the ecology of free-roaming dogs in urban environments and will be applicable to strategies aimed at promoting animal welfare and preventing the dissemination of zoonoses.

## Introduction

Domestic dogs (*Canis lupus familiaris*) are ubiquitous in all societies and cultures by virtue of the numerous benefits they undoubtedly afford to humans (1, 2). While canine populations are mainly domiciled and restrained, they may include animals that are unrestricted in their access to urban and rural areas (3, 4). The free circulation of unrestricted dogs represents a major challenge in the control of numerous zoonoses (5, 6). Rabies, for example, is responsible for around 59,000 human deaths a year and is predominantly transmitted by dog bites (7). In visceral leishmaniosis, free-roaming dogs are at higher risk of becoming infected and act in the disease dispersion (8). Furthermore, unrestricted dogs impose a substantial risk to wildlife and human safety through attacks, biting incidents, and interactions with road vehicles (9, 10).

An understanding of the factors associated with the distribution and movement patterns of free-roaming dogs in the environment is crucial in developing control actions and identifying the spreading potential of diseases (11). Free-roaming animals tend to assemble in regions with greater availability of food resources (12, 13), particularly in environments that support survival and maintenance by providing shelter, water from natural or artificial sources and surroundings with different degrees of vegetation (14, 15).

Another factor that affects the roaming and clustering patterns of unrestricted dogs is the influence of sexual stimuli. In this context, surgical sterilization, both spaying and neutering, is often used in an attempt to control the canine population, to reduce wandering and to modify undesirable behavior (16, 17). Limiting the movement of dogs is claimed to be in the interest of public safety and is certainly important in controlling and preventing the spread of infectious diseases, particularly during outbreaks (18). However, few studies have evaluated the effect of sterilization on canine behavior, especially in the case of free-roaming dogs, while the results that have been reported are often controversial or inconclusive (19, 20). In the present work, we have analyzed the influence of gender, sterilization, land cover and the presence of commercial food outlets on the spatial distribution of free-roaming dogs through capture/recapture of animals that had been submitted or not to surgical sterilization. The results of the study will extend our knowledge regarding the roaming areas and clustering behavior of free-roaming dogs in urban settings.

## Materials and Methods

### Ethics Statement

The study was approved by the Research Ethics Committee of the Universidade Federal de São João Del Rei (UFSJ; protocol no. 24/2010) and conducted according to the guidelines issued by the Conselho Nacional de Controle e Experimentação Animal (CONCEA).

### Study Areas and Data Collection

The investigation was performed in two urban areas in the municipality of Divinópolis, Minas Gerais, southeastern Brazil, that shared similar environmental and socioeconomic characteristics but were separated by a geographical barrier. Area **A** (the control area) was 1.35 × 10^6^ m^2^ in size, while area **B** (the intervention area) comprised 1.14 × 10^6^ m^2^ and was targeted for sterilization of free-roaming dogs as described in our previous report (3). Two members of the research team performed capture and recapture of free-roaming dogs (defined as animals not accompanied by an owner) with the aid of snacks. Most dogs walked up to the researchers and were attached to a halter. About 10% were captured using poles with nooses or with the help of residents. Dogs were taken to a specially adapted vehicle, where remained caged. There were seven sampling efforts conducted at 2-monthly intervals between 2012 and 2014. Sampling was carried out during a morning period. The vehicle followed the same route each catch effort and covered all streets of the designated areas. In Area A, captures were carried out in the first week of the collecting months, while in Area B, the captures took place in the second week of the same month. The location of each capture event was georeferenced using the global positioning system (GPS) and the captured animal was taken to the Health Surveillance Reference Center of Divinópolis for assessment by veterinarians.

When captured for the first time, dogs of both areas were microchipped to facilitate recognition in future recaptures, while all dogs captured in area **B** were submitted additionally to surgical sterilization. Dogs captured for the first time in a later sampling period were surgically sterilized. In all capture efforts new dogs were identified; therefore, sterilization procedures were conducted in all the seven samplings.

Following these procedures, animals were transported to and released at their original capture site. Each animal received an identification code and its details were recorded in a Microsoft Excel 2013 spreadsheet.

### Home Range of Free-Roaming Dogs

Home range (HR; defined as the area in which an animal lives and moves on a regular basis) values of dogs that had been captured at least three times were calculated using the minimum convex polygon (MCP) method (21) with the inclusion of 95% of recorded points and the exclusion of 5% of peripheral observations (22). Duplicated locations were also excluded in order to generate a convex polygon with the smallest area but containing all points. All procedures were performed with the aid of ArcGIS software version 10.5 loaded with the Home Range Tools package (23).

### Land Cover and Geoprocessing

Land cover in areas **A** and **B** was determined from aerial photographs of the region collected by the Landsat-8 satellite on 9 July 2015. The date of acquisition of the photographs was selected because it was close to the sampling dates of the study and the images exhibited adequate quality. Aerial images were submitted to orthorectification to eliminate distortion and subsequently stratified according to the normalized difference vegetation index (NDVI) (24, 25) cut-off points (CP) in order to distinguish the boundaries for each type of land cover according to: high-density vegetation zones (CP 0.06–0.12), transition zones with medium-density vegetation (CP 0.13–0.25) and urbanized zones with absence of vegetation (CP 0.25–0.40). The NDVI-based land cover zones were plotted on maps of the study areas and the location of animals distributed among the three strata. In addition, 22 commercial food outlets were georeferenced during the sampling period and their locations overlaid on the maps. Analysis of spatial data was performed using QGIS software (https://qgis.org/en/site/) version 2.18.25.

### Statistical Analysis

The HR areas were described in terms of global mean ± standard deviation (SD), median, and interquartile range (IQR). In order to analyze the effect of sterilization on the spatial distribution of dogs, median values of HR in areas **A** and **B**, along with those of male and female dogs independently, globally and between the areas, were compared using the Mann-Whitney test at the 5% significance level with the aid of R software (https://www.r-project.org) version 3.5.

The nearest neighbor distance approach was used to analyze the spatial distributions of animals and to measure spatial relationships (26, 27). The spatial densities of dogs throughout the study period, at each capture/recapture event, and for males and females separately, were determined using the Kernel technique with the aid of ArcGIS software (https://www.arcgis.com) version 10.5. The search radius was set at 100 m and the quantile normalization method was adopted as the most appropriate statistical tool for visualization of the results. The possible spatial correlation between the distribution of dogs and the locations of commercial food outlets was analyzed using Ripley's bivariate K function (28) with the aid of R software version 3.5. According to this technique, the null hypothesis of complete spatial independence cannot be rejected if the curve describing the distribution of dogs as a function of distance from a food outlet is contained inside the envelope of confidence (29).

## Results

A total of 441 captures/recaptures, involving 270 free-roaming dogs, were performed during the seven sampling efforts carried out during the study period. The proportions of animals captured in relation to the estimated population abundance (3) were 80.4% in area **A** and 61.1% in area **B**.

The highest number of captures occurred in area **A** (59.3%), while the highest proportion of recaptures took place in area **B**. In both study areas, the number of captured males was higher than that of females. In area **B**, 70 male and 40 female dogs were sterilized (Table 1). Fifty-four dogs were captured/recaptured in at least three sampling efforts, comprising 26 dogs in area **A** and 28 in area **B**, with males being most frequently recaptured and accounting for 62% in area **A** and 54% in area **B**.

**Table 1:** Number of free-roaming dogs captured/recaptured during seven sampling efforts performed between 2012 and 2014 in Divinópolis, Minas Gerais, Brazil.

**Study area**	**Captured dogs individually**			**Recaptured dogs** ***n* (%^**a**^)**
	**Males**	**Females**	**Total**	
A (control area)	90	70	160	64 (40.0)
B (intervention area)	70	40	110	50 (45.4)

a *Proportion of dogs recaptured at least once in relation to the total number of dogs captured in each area*.

Application of the MCP method to dogs that had been captured/recaptured at least three times generated a global mean HR of 448 m^2^ (SD = 1,398 m^2^). The smallest HR in both areas was 1 m^2^, whereas the largest were 7,868 m^2^ for area **A** and 3,704 m^2^ for area **B** (Supplementary Material). The median HR and IQR values for free-roaming dogs captured/recaptured in areas **A** and **B** are shown in Table 2. There were no significant differences in HR values between areas **A** and **B** (*P* = 0.70), between males from areas **A** and **B** (*P* = 0.45), between females from areas **A** and **B** (*P* = 0.61), between males and females from area **A** (*P* = 0.89), between males and females from area **B** (*P* = 0.28) or between males and females globally (*P* = 0.35).

**Table 2:** Home range (HR) values of dogs captured/recaptured at least three times in two areas of Divinópolis, Minas Gerais, Brazil, within the period 2012–2014.

**Study area**	**HR (m**^****2****^**)** **Median values (Q1–Q3 range)**		
	**Males** ***N* = 31**	**Females** ***N* = 23**	**Both genders** ***N* = 54**
A (control area) (*N* = 26)	18 (7–298)	61.5 (8–118)	27 (8–131)
B (intervention area) (*N* = 28)	7 (3–99.5)	86 (6–153)	28.5 (4–148)
Global values	15 (3.5–137)	64 (7.5–141.5)	28 (5–142)

*Q1, first quartile; Q3, third quartile; N, Number of dogs*.

**Figure 1:**
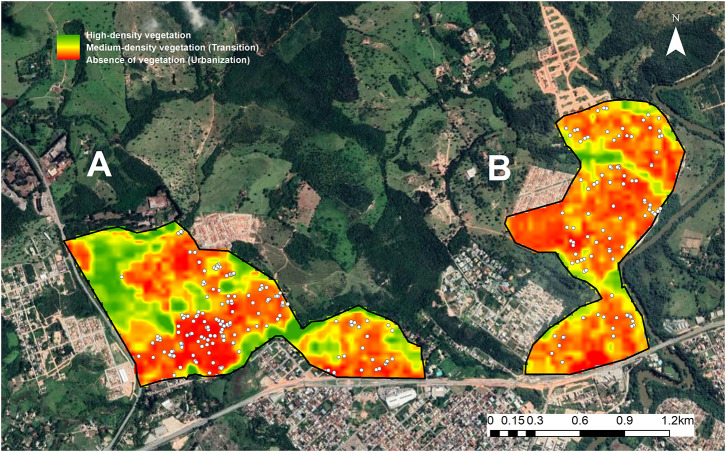
**(A,B)** Land cover and spatial distribution of free-roaming dogs in two areas of Divinópolis, MG, Brazil, studied during the period 2012 to 2014. Zones with high-density vegetation are shown in green, transition zones with medium-density vegetation in yellow and orange, and urbanized zones with absence of vegetation in red. The white dots represent dog capture locations.

Animals were distributed in clusters in all seven sampling efforts and the clustering was statistically significant (*P* < 0.0001). According to NDVI values, urbanized zones with absence of vegetation were predominant (63.6%) in the study region, followed by zones with a high-density of vegetation (29.2%) and transition zones (7.2%). With regard to the distribution of free-roaming dogs, 71.7% were captured in zones with absence of vegetation, 28.3% in transition zones and none in the high-density vegetation zones (Figure 1).

**Figure 2:**
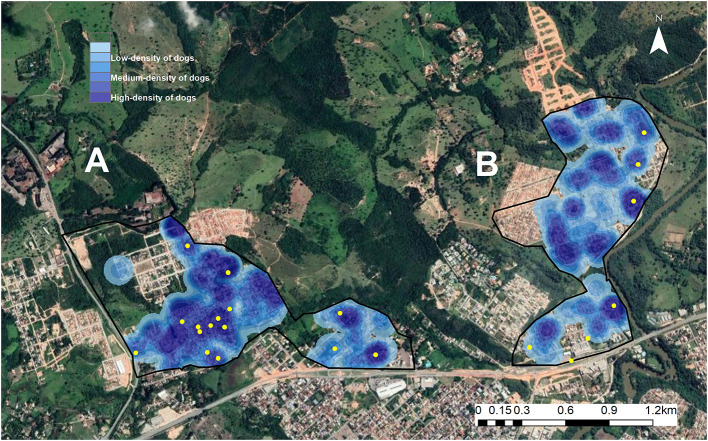
**(A,B)** Spatial analysis of two areas of Divinópolis, MG, Brazil, showing the locations where free-roaming dogs congregated. Areas with a high-density of dogs are shaded dark blue, medium-density areas are mid blue and low-density areas are light blue. Yellow dots indicate the locations where commercial food outlets were located.

**Figure 3:**
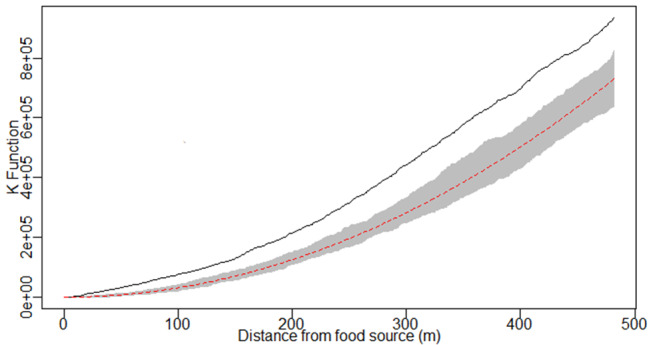
Ripley's bivariate K function. The dashed red line represents the theoretical Poisson K-function, the gray band represents the envelope of confidence, while the unbroken black line represents the observed K function and reveals a positive spatial correlation between the distribution of dogs and commercial food outlets.

Although the regions with the highest concentrations of male and female dogs were similar, males generally concentrated in fewer locations than females (Supplementary Material). According to Kernel density mapping, dog density was high in 40.7% of the study areas, medium in 22.5% and low in 36.8% (Figure 2). The dog density profile was compared with the distribution of commercial food outlets in the study areas in which 63.3% (14/22) of the establishments were located in high-density zones, 31.2% (7/22) were in medium-density zones and 4.5% (1/22) were in low-density zones. According to the K function, there was a positive spatial dependence between dog distribution and the location of commercial food outlets (Figure 3).

## Discussion

The aim of the present study was to estimate the roaming areas of free-roaming dogs in urban regions of a medium-size town in southeastern Brazil and to analyze the influence of various factors on the spatial distribution of these animals. Such information is important for tackling endemic zoonotic diseases and devising suitable population control and animal welfare programs.

The estimated global mean HR (448 m^2^) was similar to that reported by Daniels (30) for free-ranging dogs in Newark, NJ, USA, but lower than the values cited in studies carried out in rural areas or in communities located in regions encompassing a high-density of vegetation (20, 31–35). It is known that the movement of free-roaming dogs depends on specific characteristics of the geographical settings and on the availability of food (20). In densely populated urban centers, exemplified by the areas of the present study, free-roaming dogs do not have to travel large distances in order to find food, a situation that is similar to that reported for non-domesticated carnivorous species that adapt to urban areas and human-modified habitats (36). Furthermore, disparities between sampling times and methodologies employed in the estimation of HRs in previous studies may have contributed to variations observed in the published results. In the present study, samplings were performed in the mornings when canine activity is high (37) and HR was estimated using the MCP method, which may overestimate the measurement (31). Nevertheless, it is possible to state that the HR pattern of free-roaming dogs was consistent and characterized by low mobility within the study areas. This conclusion is reinforced by the observation that the dogs tended to group in similar regions in all sampling efforts.

Another factor that may affect the estimates of HR of free-roaming dogs is the follow-up time of the animals. In the present study, the 54 dogs for which HR values could be estimated showed follow-up times between 6 and 14 months. Despite the protracted follow-up times, which are somewhat longer than those reported in the literature, the HR areas of the free-roaming dogs were small and this may be associated with the care provided by residents of the study areas. Indeed, in these areas there was a predominance of community dogs receiving food support from absent owners, who allowed total freedom to their animals, and from caring neighbors (3). Such dogs presented a limited circulation range, since they did not need to travel far to find food and shelter, and enjoyed a greater chance of survival (38) in as much as they were captured/recaptured several times and, thereby, included in the estimates of HR. On the other hand, the variability in HR values was high indicating the existence of population groups with different mobility patterns as reported in the literature (31, 32, 39). Considering that dogs with larger HR areas bestow a greater probability of disease transmission, efforts must be made to ensure that such animals are included in zoonoses prevention and control actions (32, 40).

Some authors have reported that the HR areas of free-roaming male dogs are larger than those of their female counterparts (20, 31) and that owned males have higher odds of roaming (41) although the results are controversial (42). Our study indicated the opposite pattern, but without statistical significance. Apparently, dog owners generally prefer male animals (38, 43), thus it is possible that free-roaming females in the study areas were less cared for, or supervised by local residents and, therefore, had to travel further in search of food. In future studies, it would be important to determine whether the mobility patterns of male and female dogs in urban regions are consistent, since this aspect may be relevant for the establishment of animal welfare and population control measures as well as actions against transmission of zoonoses (20).

Our finding of a spatial correlation between the distribution of free-roaming dogs and the presence of commercial food outlets is similar to the situation reported by Dias et al. (12) in which stray dogs on a university campus tended to congregate in locations near to the restaurant area. These results support the hypothesis that food availability strongly influences the spatial distribution of free-roaming dogs and is the driving force in areas of high population density (3, 44). For this reason, it has been suggested that the population of free-roaming dogs could be controlled by limiting access to food (45), with reductions being imposed gradually to avoid aggression among the animals (46). However, curbing food availability would be difficult to implement in a socio-cultural context since the measure is ethically questionable and could encourage free-roaming dogs simply to seek out new food sources in alternative areas. It is likely that promotion of animal welfare programs and of responsible pet ownership would produce results that are more effective in the long term (3, 47).

The dog clusters identified in zones without food outlets may be explained by factors not directly analyzed in our survey, but identified in the study areas, including the occurrence of community-supplied fixed dog feeders and the existence of communities more tolerant to the presence of dogs (13). Alternatively, other factors may be extant that are intrinsic to the social and territorial organization of the animals (48).

Growth in the human population may influence the augmentation of unrestricted canine populations (49). In the present study, free-roaming dogs predominated in zones with absence of vegetation and in transition zones, but such animals were not observed in regions with a high-density of vegetation. Ordeñana et al. (50) reviewed the effects of urbanization on the distribution of carnivores and reported a positive relationship between the abundance of domesticated dogs and the intensity of urbanization. Thus, changes in the environment that result in urbanization can facilitate animal clustering and transmission of diseases. Furthermore, Brookes et al. (11) have demonstrated that infectious diseases can spread rapidly between canine networks. In the present study, we have identified a tendency of dogs to form groups in zones with less vegetation and those with food outlets, and suggest that fragmentation of the canine population may represent a viable strategy to reduce the probability of transmission of infections.

We have investigated for the first time the influence of sterilization on the distribution of free-roaming dogs by comparing an area in which male and female dogs were submitted to surgical sterilization with another area in which animals were not subjected to intervention. Our results revealed that sterilization did not produce significant alterations in the HR areas of free-roaming dogs. A similar result was reported by Garde et al. (19) who followed-up male dogs that had been chemically or surgically neutered in the small town of Puerto Natales, Chile, and observed no reductions in HR area, sexual activity or aggressive behavior of the animals. Sparkes et al. (20) assessed the effects of gender and reproductive state on short-term activity patterns and contact rates of free-roaming dogs in an Australian indigenous community. These researchers reported that contacts between intact females and neutered males were more frequent than those involving spayed females and intact males. Various hypotheses have been put forward to explain the perpetuation of sexual activity, contact and roaming behavior of sterilized free-roaming dogs, including the breakdown of dominance hierarchy (20), the continuance of sexual behavior in neutered males despite the possibility of effective copulation, and the increased appetite of castrated dogs (16, 19). Studies of the efficacy of sterilization for the modification of behavior of confined dogs have produced results that are contradictory and unreliable owing to methodological limitations (16). In the light of data presented herein, as well those in the literature regarding free-roaming and confined dogs, we contend that there is no consistent evidence to support the benefits of sterilization in reducing dog mobility. On the other hand, capture, neuter, and return programs can be beneficial in stabilizing the population, decreasing public health risks, and improving the welfare of dogs (51).

The present study was subject to certain limitations regarding the estimation of HR, principally because only a small number of dogs were captured/recaptured more than three times, thereby diminishing the precision of HR measurements and the power of statistical tests. Thus, we choose to discuss the values estimated and not only the statistical significance. Moreover, since the dogs were sterilized for a varying period of time, there may not have been enough time for the effects of sterilization to occur in all animals. Captured dogs were not fitted with real-time GPS trackers, hence it was not possible to calculate the average distance traveled or to use other techniques to calculate HR. Nevertheless, application of the MCP method, which shows good accuracy in small sample cases (52), generated HR values that were sufficiently precise to allow the effects of sterilization to be evaluated. Finally, the observed pattern of dog behavior was based on samplings performed in the mornings and may not be representative of other periods of the day. For example, dogs are generally less active during the night time (37), although male dogs may roam more than females at night and aggressive behavior for territorial dominance is often intensified (53).

## Conclusions

The mean HR values of free-roaming dogs in urban settings in a medium-size town in southeastern Brazil, were smaller than those previously reported for areas with predominantly rural characteristics. However, HR areas were highly variable indicating the existence of population groups with different roaming behavior and a predominance of animals with small HRs. In general, females presented higher HR values compared with males, although this may reflect specific characteristics of the study area such as the prevalence of community dogs cared for by local residents. The spatial distribution of free-roaming animals was positively associated with the degree of urbanization and the presence of commercial food outlets, implying that roaming behavior and clustering were predominantly influenced by the availability of food. Sterilization did not reduce the HR areas of free-roaming dogs, verifying that the mobility of such animals is multifactorial.

The results reported herein increase our understanding of the factors associated with the spatial distribution of free-roaming dogs in urban settings, knowledge of which is important in controlling the canine population, promoting animal welfare actions and decreasing the transmission of zoonoses. The estimated HR values obtained will also be useful for the construction of predictive mathematical models. Information regarding free-roaming dogs would be greatly enhanced by conducting in the future longer surveys in larger urban centers with additional information on covariates describing physical aspects of dogs that could affect their HR and involving samplings at different times of the day and, ideally, with real-time GPS tracking of animals to establish the locations of community feeders and preferred shelters.

## Data Availability Statement

All datasets generated for this study are included in the article/Supplementary Material.

## Ethics Statement

The animal study was reviewed and approved by Research Ethics Committee of the Universidade Federal de São João Del Rei.

## Author Contributions

SM, DC, and VB organized the database. SM, DC, GW, CS, and VB performed the statistical analysis. SM and VB wrote the first draft of the manuscript. All authors contributed to conception and design of the study, wrote sections of the manuscript and contributed to manuscript revision, read, and approved the submitted version.

## Conflict of Interest

The authors declare that the research was conducted in the absence of any commercial or financial relationships that could be construed as a potential conflict of interest.
