# Supplementary material for: Effects of Gender, Sterilization, and Environment on the Spatial Distribution of Free-Roaming Dogs: An Intervention Study in an Urban Setting
Source: Front Vet Sci. 2020 May 27;7:289. doi: 10.3389/fvets.2020.00289 (PMC7266977; doi:10.3389/fvets.2020.00289)

Supplementary Material

Spatial distribution of free-roaming dogs in a medium-size town in southeastern Brazil showing: a) the seven samplings; b) the distribution of males and females and c) the individual home-ranges of males and females.

1. Spatial distribution of free-roaming dogs in a medium-size town in southeastern Brazil showing: **1)** first sampling; **2)** second sampling; **3)** third sampling; **4)** fourth sampling; **5)** fifth sampling; **6)** sixth sampling; and **7)** seventh sampling. Kernel density spots are shown as dark blue (high density), mid blue (medium density) and light blue (low density).

*
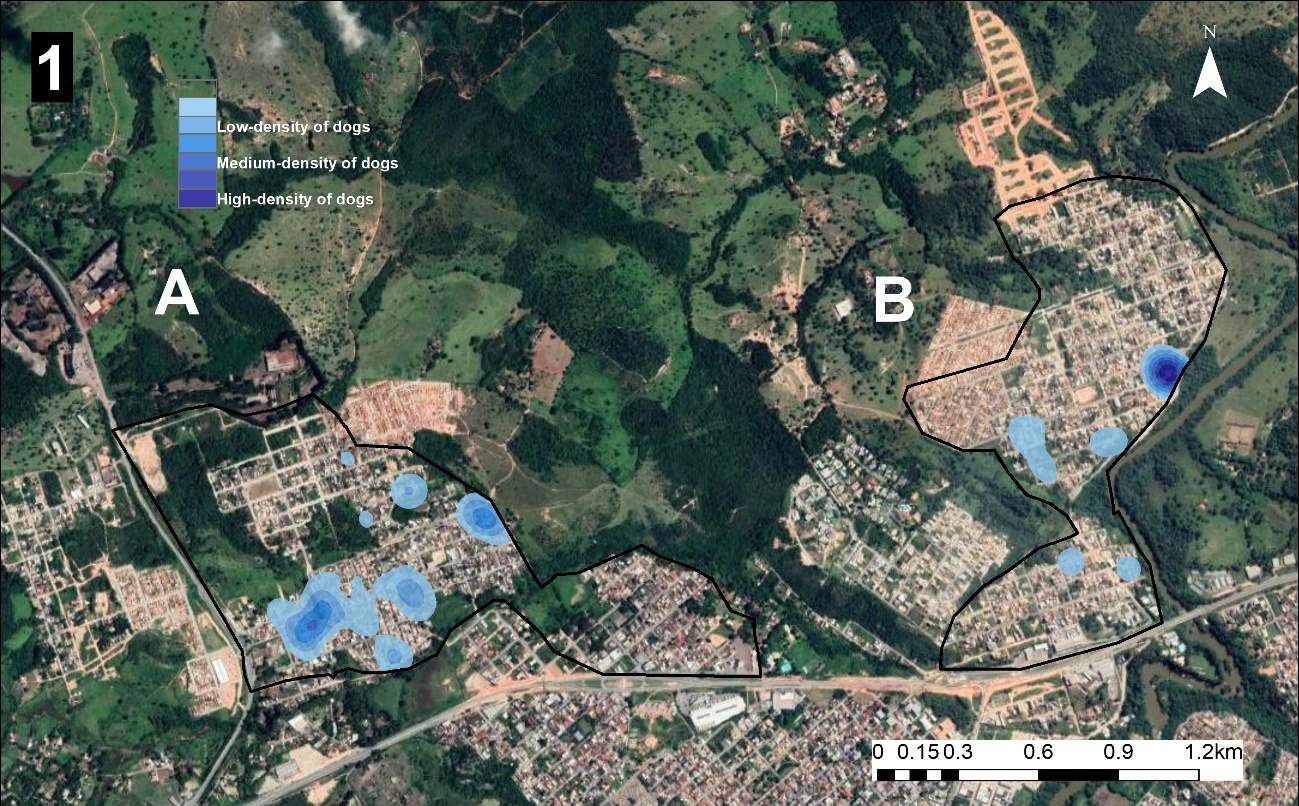
*

*
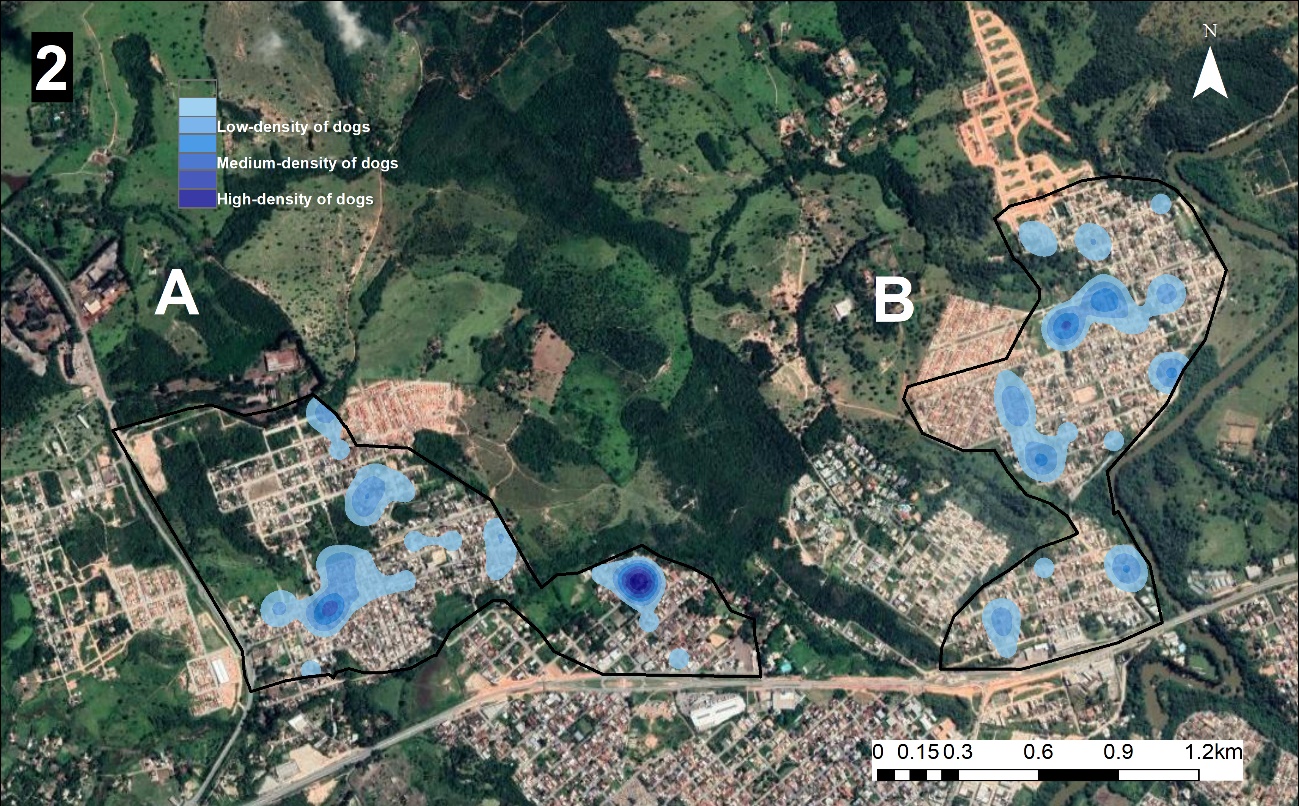
*

*
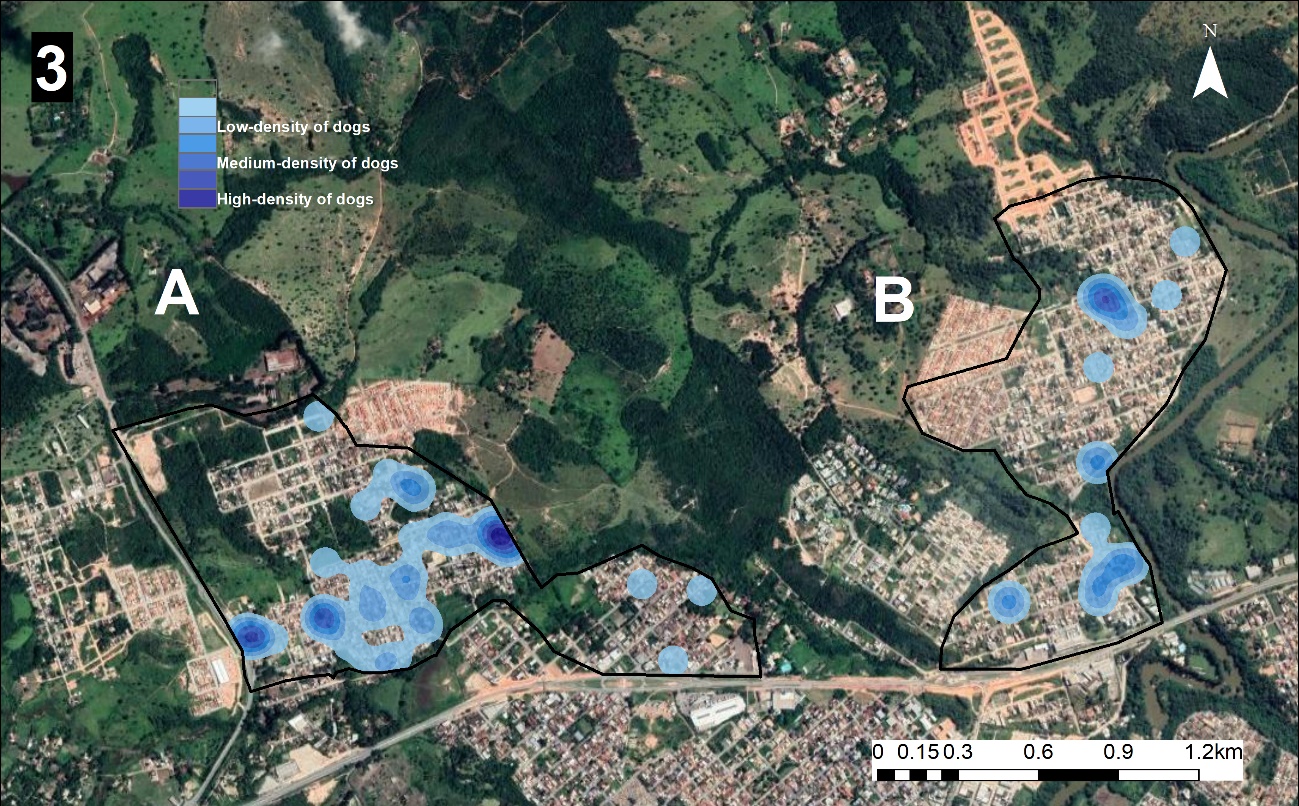
*

*
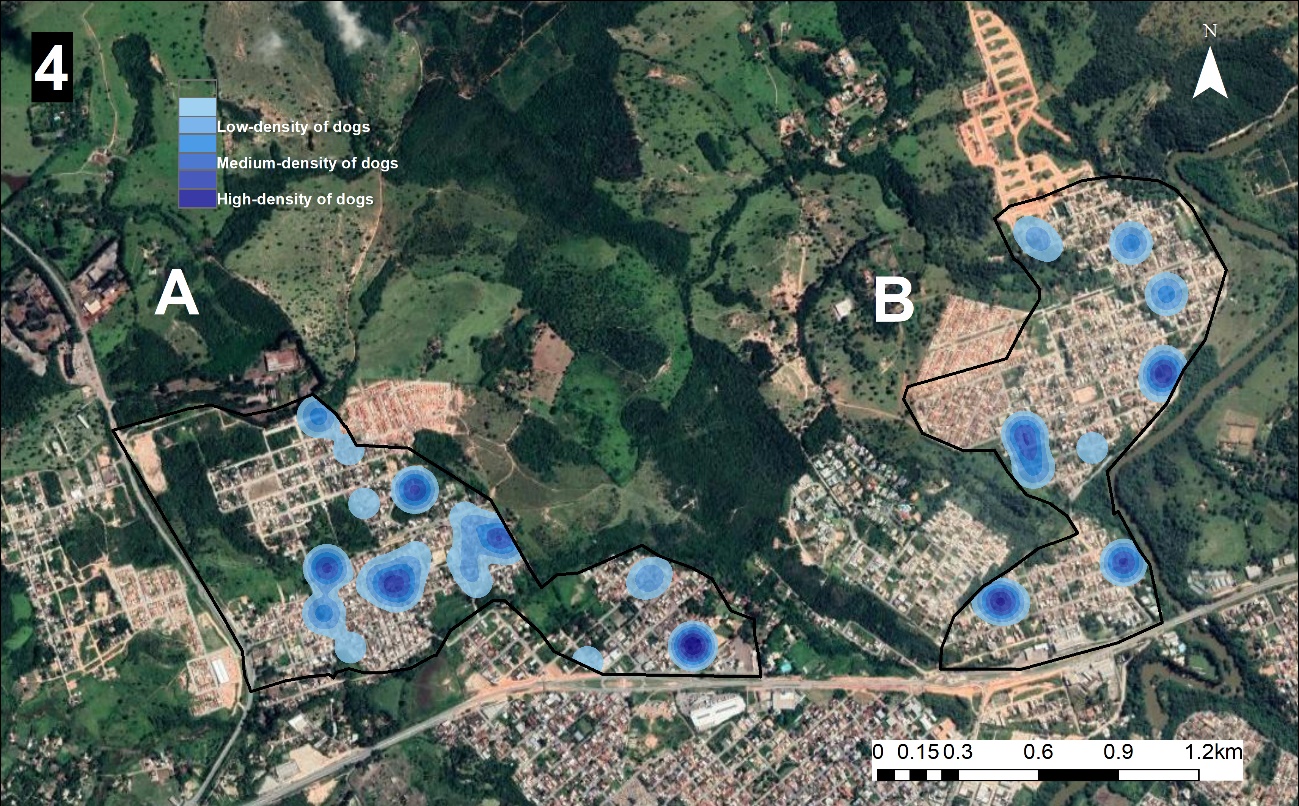
*

*
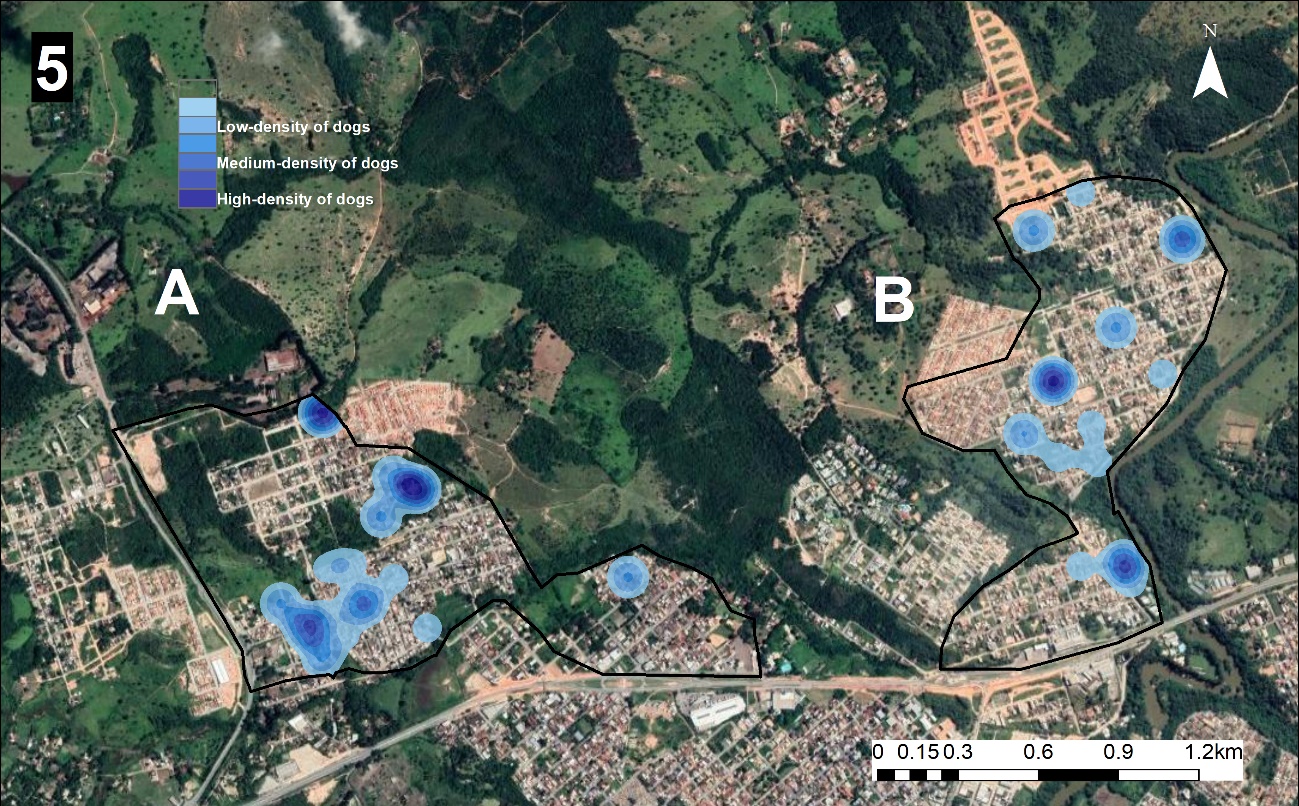
*

*
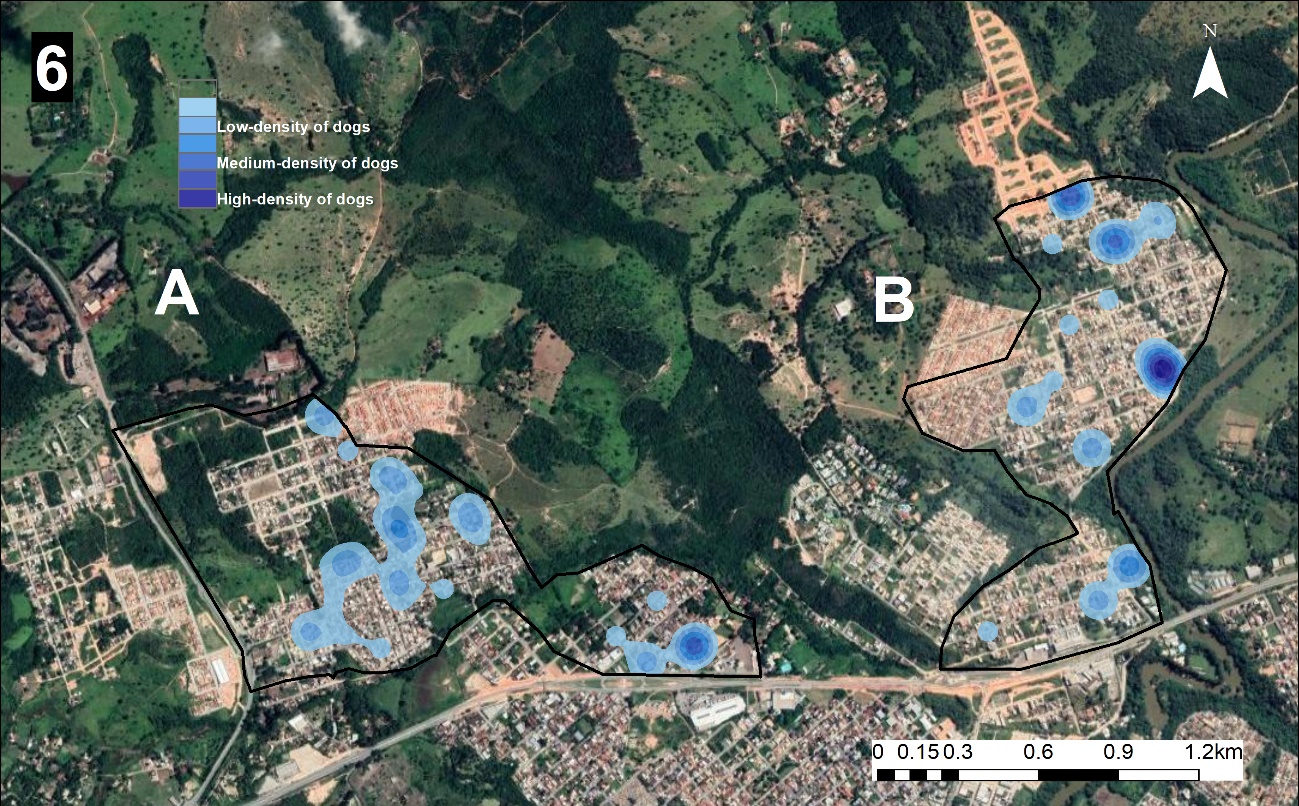
*

*
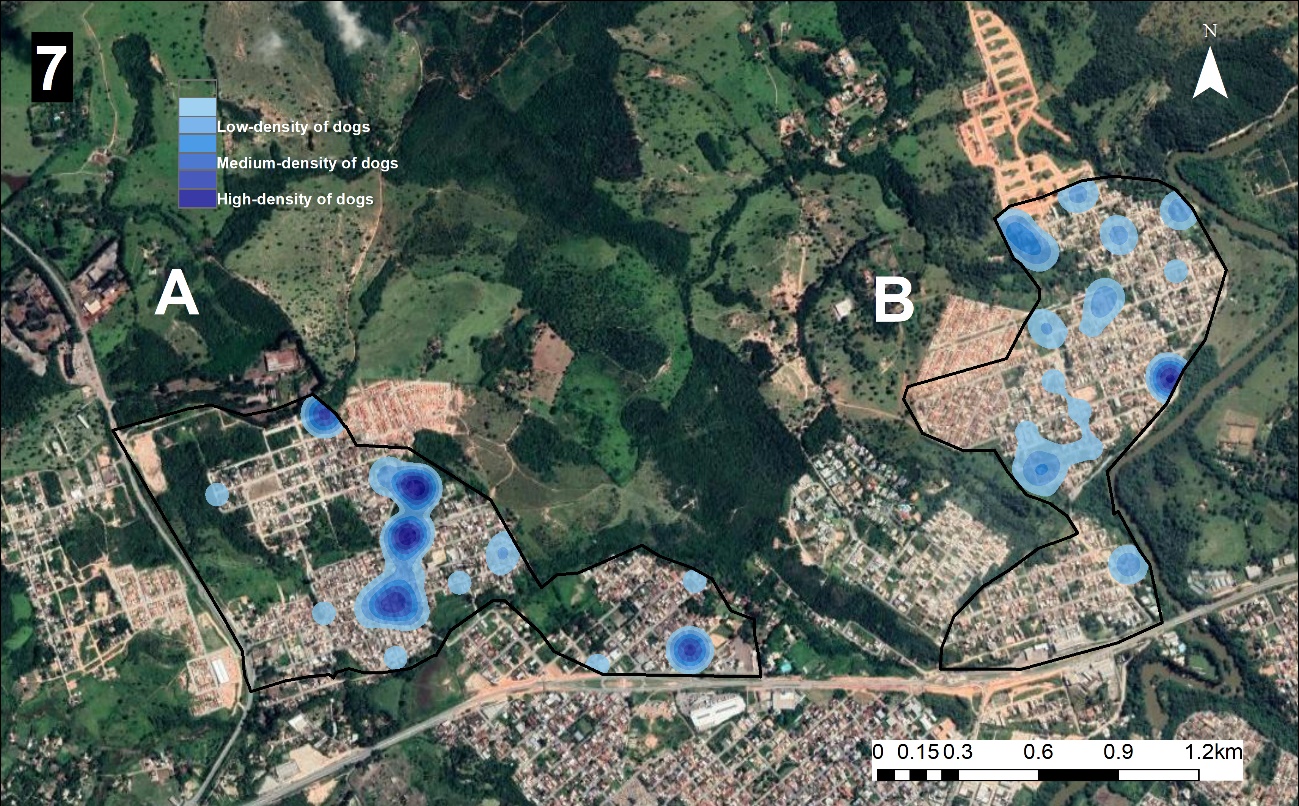
*

1. Spatial distribution of free-roaming dogs in a medium-size town in southeastern Brazil showing: 1**)** females; and **2)** males. High density spots are shown in dark blue, medium density spots in mid blue and low density spots in light blue.


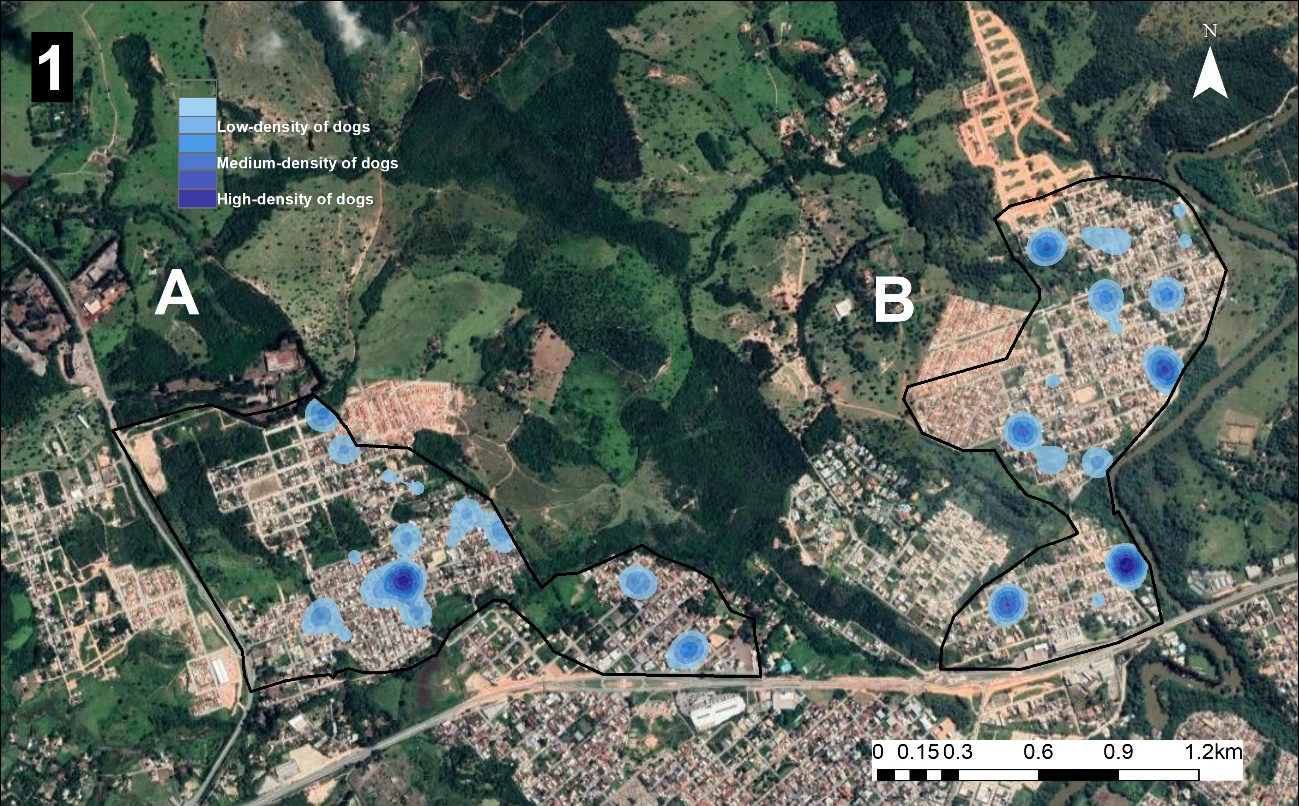


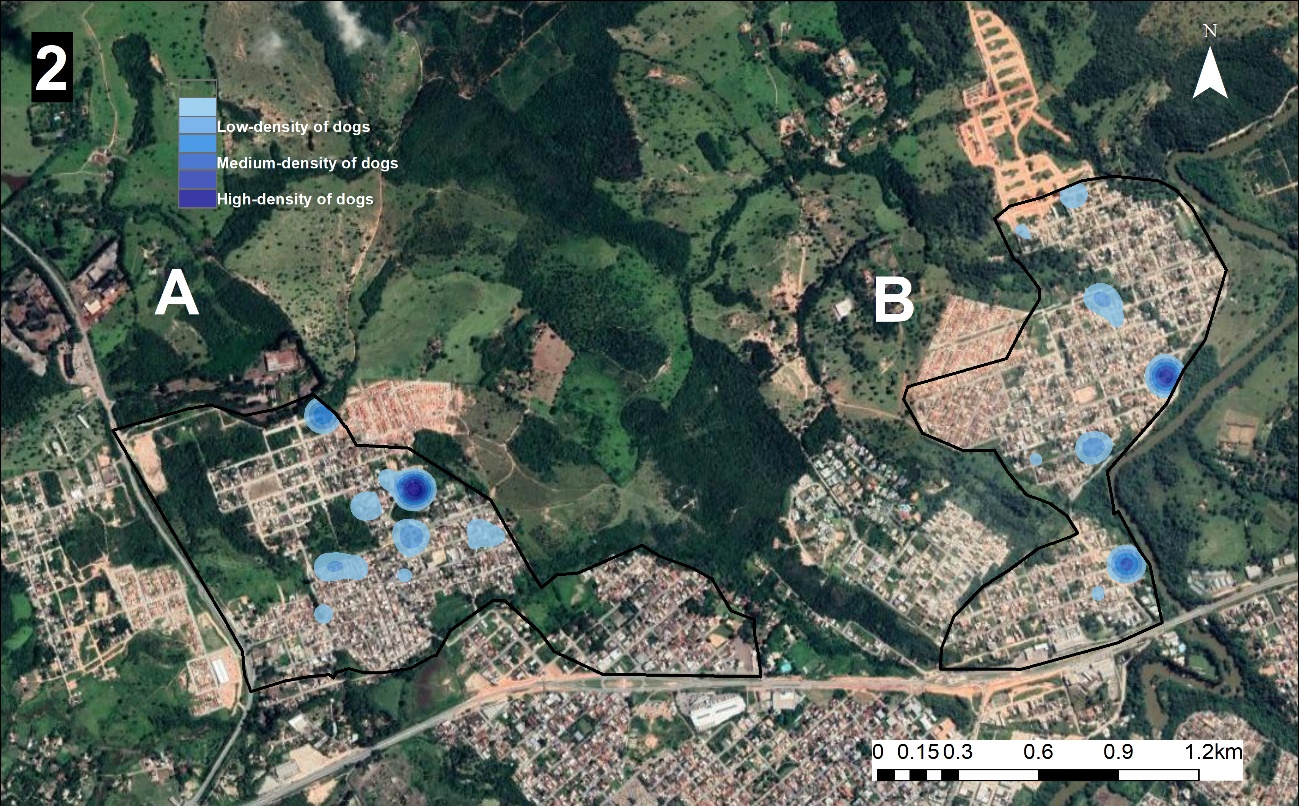


1. Home-ranges of free-roaming dogs in a medium-size town in southeastern Brazil: Yellow markings indicate the most likely home-ranges for males and red markings indicate the most likely home-ranges for females.


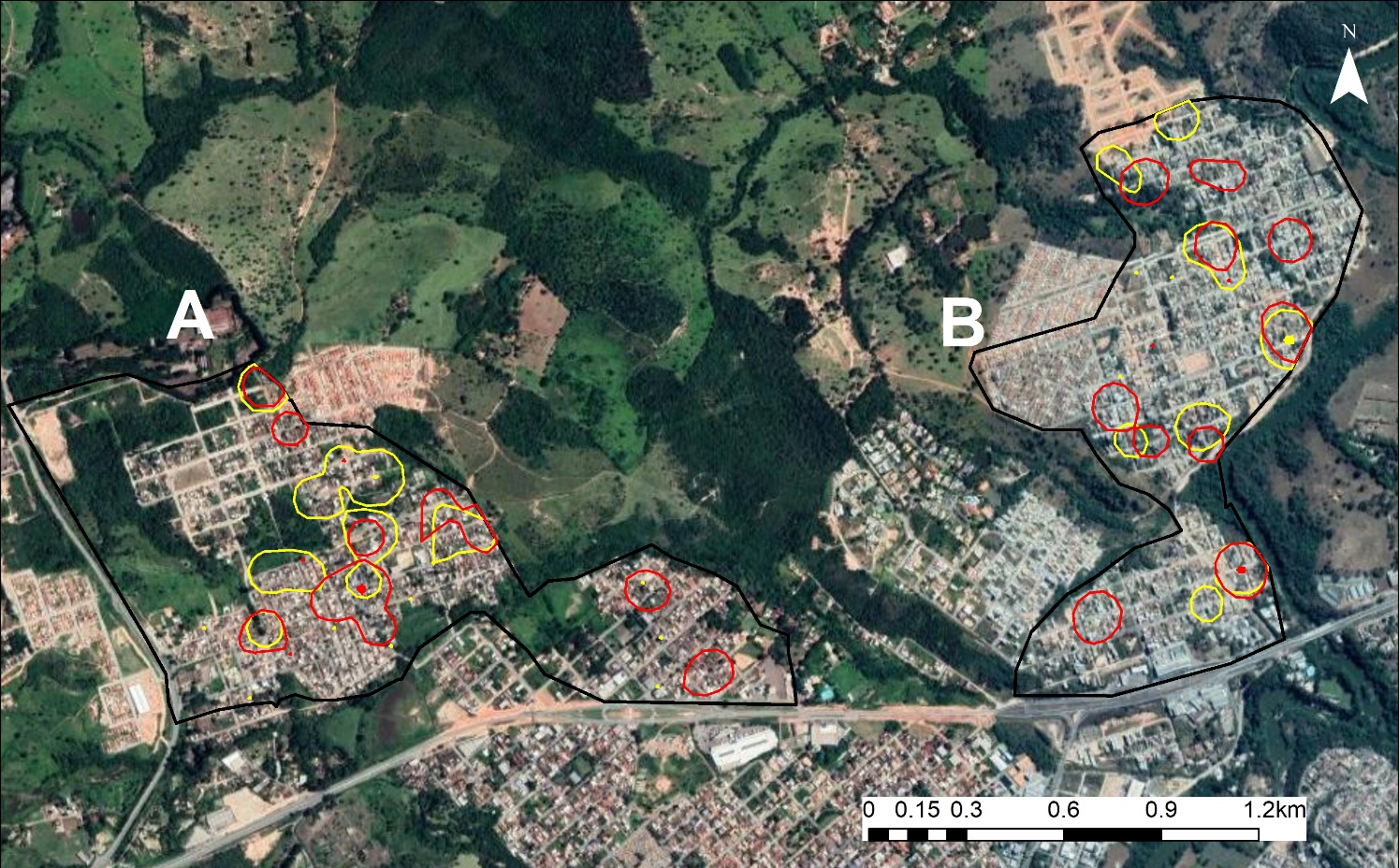

Supplement: Supplementary file 2 [file Data_Sheet_2.docx]
